# Verifying the usefulness of the theory of planned behavior model for predicting illegal use of online content: the role of outcome expectancies and social loafing

**DOI:** 10.1186/s40359-022-00978-3

**Published:** 2022-11-12

**Authors:** Yulee Choi, Kyung Hyun Suh

**Affiliations:** 1 Department of Interdisciplinary Arts, Sahmyook University, Hwarang-ro 815, Nowon-gu, Seoul, 01795 South Korea; 2 Department of Counseling Psychology, Sahmyook University, Hwarang-ro 815, Nowon-gu, Seoul, 01795 South Korea

**Keywords:** Piracy, Online content, Theory of planned behavior, Outcome expectancy, Social loafing

## Abstract

**Background:**

Currently, laypeople can earn profit by producing content; therefore, it should be noted that the unauthorized use of intellectual creations or possessions can cause legal issues and undermine the producers’ desire to create. This study verified the usefulness of the theory of planned behavior (TPB) model for predicting the illegal use of online content among South Korean college students and examined the roles played by outcome expectancies and social loafing in this model.

**Methods:**

The participants, 369 male and female Korean college students, were aged between 18 and 31 years (M = 22.12, SD = 2.33). We measured the illegal use of online content and the factors of the TPB model, as well as outcome expectancies and social loafing with regard to participants’ illegal use of online content. Correlational analysis, stepwise regression analysis, path analysis for the TPB model, and analyses of the moderated model were performed using SPSS and AMOS.

**Results:**

All TPB factors, outcome expectancies, and social loafing were positively correlated with the illegal use of online content. Stepwise regression analysis showed that intention, social loafing, outcome expectancies, and perceived behavioral control were significant predictors of the illegal use of online content. A TPB model, including a direct path from perceived behavioral control to behavior, was validated to analyze the illegal use of online content. This model was found to be moderated by outcome expectancy.

**Conclusion:**

This study suggests that the TPB is useful for predicting the illegal use of online content and that outcome expectancies and social loafing also play an important role in the illegal use of online content among college students. The findings of this study provide useful information for future research and could aid in preventing illegal online content use among adolescents and young adults.

## Background

When copyright law was enacted in England approximately 100 years ago, few people thought it would become an important social issue [1]. Despite the existence of such laws, the issue of content use without permission has persisted; more recently, ordinary laypeople, rather than professionals, have earned profit through online content production, which can create various problems if such people use intellectual creations and property without permission [2]. These issues have become imperative in the current era when digital creations are traded and shared over the Internet [3]. There are some concerns about it being a threat to the future of the Internet, as there are some legal prohibitions on the sharing of online content on social network services (e.g., Facebook in Europe). However, it is necessary to protect creative industries [4]. If copyright is not protected with regard to the production of online art content, there is no motivation for content creation and development.

If there is no proper prohibition on illegal copying of digital content, creators’ profits will drop sharply [5]. The illegal use of digital content not only reduces creators’ efforts and producers’ motivation to develop new content but also abuses market regulations governing digital content distribution [6, 7]. Moreover, some analysts have stated that the illegal use of online content can threaten the national economy [8]. Therefore, it is important to control illegal online content usage. However, previous research has found that there could be differences in attitudes toward digital piracy and illegal online content use between content users and content creators or content industry workers [9]. If so, it is possible to establish policies and control strategies for prohibiting illegal online content use only when the psychological characteristics of consumers who use online content illegally are investigated. Thus, this study explores the psychological characteristics of online content consumers regarding the illegal use of such content.

### Theory of planned behavior and illegal online content use

This study attempted to verify a model to explain illegal online content use among college students. We selected the theory of planned behavior (TPB) as a theoretical model for explaining such illegal content use. TPB was proposed by Ajzen and has been investigated as a theoretical model for predicting various types of human behavior [10–14]. The TPB extends the theory of reasoned action, which includes the behavioral intention between attitudes or subjective norms and human actions due to the low accountability of attitudes toward individuals’ behaviors [15]. Ajzen added perceived behavioral control (a concept similar to self-efficacy) as a variable affecting behavioral intentions, in addition to attitudes and subjective norms [10]. Several studies have shown that perceived behavioral control (rather than attitudes, subjective norms, and behavioral intentions) has the strongest impact on behaviors [16–18], thus indicating the TPB’s validity. Further studies on this subject are required.

TPB has also been frequently studied in relation to unethical and illegal behaviors [19]. TPB has often been verified as a theoretical model for predicting content use behavior or digital piracy [20–24]. This theoretical model has also been explored as an expanded model that includes other variables [25–27]. However, this study attempts to verify a model that includes variables for moderating the path between attitude and behavioral intention as an expanded TPB model. Previous studies have verified the validity of these models. For example, Suh found that optimistic or present biases moderate the TPB model [14]. Furthermore, another study showed that social dilution of responsibility could moderate a TPB model for high school students’ illegal use of music sources [24]. Thus, this study also attempted to verify the usefulness of a TPB model for predicting the illegal use of online content among college students and explored some variables for moderating this model.

### Moderators of the TPB model for illegal online content use

We assumed that, even if an individual does not hold a negative attitude toward illegal online content use, if there is no expectation of the outcome of using it, the possibility of not using it will be high, and vice versa. Outcome expectancies form an important part of Bandura’s social cognitive theory; they refer to a person’s belief in the consequences of their prospective behavior [28]. Several studies have investigated outcome expectancies as a determinant variable for conducting behaviors that are difficult to practice [29]. Furthermore, as outcome expectancies play an important role in individuals’ behavioral changes [30], identifying outcome expectancies might be useful for predicting behavior.

Studies have also empirically confirmed that outcome expectancies are significant predictors of digital piracy and illegal use of music sources and have shown high accountability in previous studies [31, 32]. If so, we can assume that there will be no significant difference in behavioral intention of illegal use of online content if individuals do not expect any outcome from using online content illegally, although their attitudes toward illegal online content use are positive. Therefore, we hypothesized that outcome expectancies would moderate the TPB model for college students’ illegal online content use.

Although there is a type of social facilitation whereby the social situation promotes positive behaviors [33], individuals’ responsible behaviors may be reduced when they are in a crowd [34]. This phenomenon is called social loafing, in which depersonalization occurs in social situations [35] because of the following thought process: if one individual in the group fails to fulfill the relevant responsibilities, nothing significant will happen; furthermore, if that individual does fulfill the relevant responsibilities, there will be no consequential positive impact. In short, social loafing refers to the phenomenon in which individual responsibility is socially diluted.

An individual’s motivation to be socially responsible decreases in a large group and individuals become less aware of the importance of the laws or rules that must be followed [36]. For example, in regard to copyright law, a previous study showed that teenagers experiencing high levels of social responsibility dilution were more likely to use music sources illegally [32]. Conversely, it is highly likely that individuals do not intend to use online content, despite the fact that they personally prefer illegal online content. If so, an interaction between attitude toward the illegal use of online content and social loafing on the intention to use illegal online content may be significant. Lim and Suh found that social responsibility dilution could moderate the path between attitude and behavioral intention in the TPB model for high school students’ illegal use of music sources [24]. Therefore, it is necessary to identify whether social responsibility dilution (i.e., social loafing) can moderate the TPB model with regard to college students’ illegal online content use.

### Purpose and hypotheses of the study

This study aimed to examine a TPB model for predicting illegal online content use among Korean college students and to analyze whether this model was moderated by outcome expectancies or social loafing. It is not the first to verify the TPB model in relation to digital piracy; however, because it is discriminatory to examine the moderating effects of outcome expectancies and social loafing on the TPB model, it will contribute greatly to education and policy to prevent illegal online content use.

**Fig. 1:**
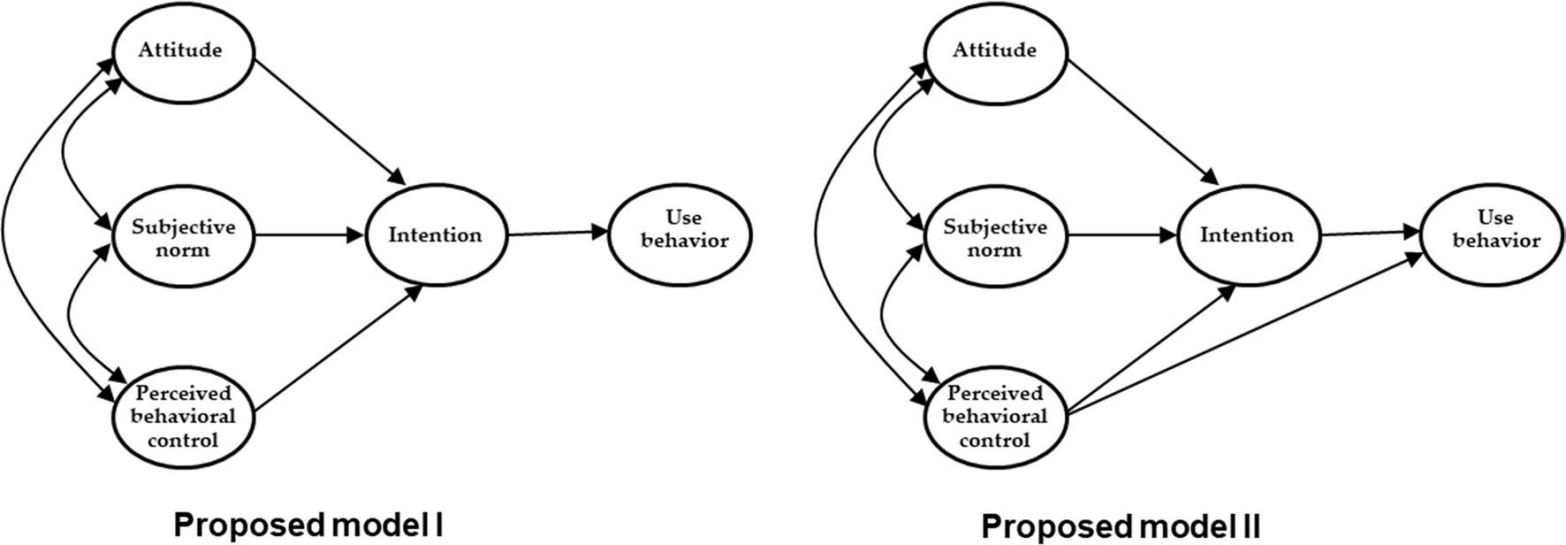
Proposed models of TPB for illegal online content use

To achieve this, we examined the following hypotheses:

- H1: There are significant relationships among TPB factors, outcome expectancies, social loafing, and illegal online content use behavior among college students.
- H2a: A model that includes only indirect paths from attitude, subjective norms, and perceived behavioral control via the intention to engage in illegal behavior regarding online content use is acceptable (Fig. 1).
- H2b: A model that includes indirect paths from attitude, subjective norms, and perceived behavioral control via intention to illegal online content use behavior, with a direct path from perceived behavioral control to use behavior, is acceptable (Fig. 1).
- H3a: Outcome expectations have a significant moderating effect on the TPB model.
- H3b: Social loafing has a significant moderating effect on TPB.

The verification of these hypotheses could provide valuable information and knowledge to prevent illegal online content use.

## Methods

### Participants

The study participants were 369 male and female South Korean college students. Data were collected by commissioning Embrain, an online survey company in South Korea. The G^*^Power 3.1 calculated that the required minimum sample size was 172 to reach the statistical conclusion based on the number of predictors, significance level (0.05), power (0.95), and effect size (0.15). However, because it should be divided into two groups of high and low according to the assumed moderators, the minimum sample size had to reach at least 344, which is twice the calculated number of 172.

In total, 183 (49.6%) were male participants, and 186 (50.4%) were female participants. Their ages ranged from 18 to 31 years, with a mean of 22.12 ± 2.33 years. Among these, 63 (17.1%) were freshmen, 81 (22.0%) were sophomores, 96 (26.0%) were juniors, and 129 (35.0%) were seniors. A total of 152 (41.2%) participants reported the humanities or social sciences as their major; 173 (46.9%) reported sciences, engineering, or medical sciences; 25 (6.8%) reported arts or sports sciences;

 and 19 (5.1%) reported other majors.

### Instruments

#### Factors of TPB

We measured the TPB model factors regarding participants’ illegal online content use with the aid of modified items from previous studies conducted in Korea [37, 38]. We have modified these items to suit the subject matter of this study. These items were developed based on Ajzen’s theory [10]. The items for attitude measured whether college students thought positively or negatively about illegal online content use (e.g., “If it is not for commercial purposes, it is okay to use content such as music sources and videos illegally”); items for subjective norms measured their perception of the social norms related to illegal online content use (e.g., “People around me think that it is okay for me to use illegal online content”); and items for perceived behavioral control measured how much control they exerted when using online content illegally (e.g., “I know how to download content illegally”). Furthermore, items of intention concerned illegal online content use (e.g., “I will use online content illegally regardless of copyright issues”). Each factor was measured using four items on a six-point scale ranging from 1 (*not at all true*) to 6 (*very true*), as a five-point scale has a neutral option that respondents may choose without much thought. Additionally, it was found that the reliability increased when a six-point scale was used rather than a four-point scale [39]. In this study, internal consistencies (Cronbach’s α) were 0.74 for attitude, 0.81 for subjective norms, 0.92 for perceived behavioral control, and 0.92 for intention.

#### Illegal use of online content

Participants’ illegal online content use was measured using modified items constructed by Lim and Suh [24]. Lim and Suh constructed items according to recent trends and previous studies [37, 40, 41], and the use of music sources was modified and used as online content in this study. This scale has five items regarding whether respondents obtain music sources and videos through illegal stream-ripping on YouTube, whether they have used illegal online content that they have not officially purchased, and whether they have ever downloaded and used it illegally through the Internet. Each item was rated on a six-point Likert scale ranging from 1 (*not at all true*) to 6 (*very true*). If the score was high, respondents were considered to show frequent illegal use of online content. In this study, Cronbach’s α for all five items was 0.80.

#### Outcome expectancies

The outcome expectancies of illegal online content use—the degree of participants’ expectation of gains from using online content illegally—were measured using items developed by Lim, with reference to items used in previous studies [32, 40, 41]. We have modified these items to suit the subject matter of this study. Four items measured whether respondents expected to save money through the illegal use of music or access new music and videos faster. Each item is rated on a six-point Likert scale ranging from 1 (*not at all true*) to 6 (*very true*); a higher score indicates a greater number of expected outcomes from illegal online content use. Cronbach’s α for the items was 0.83 in this study.

#### Social loafing

We used the Dilution of Social Responsibility Questionnaire (DSPQ) [24] to measure participants’ social loafing regarding their illegal online content use. Originally, this questionnaire measured social loafing with regard to the act of discipline or illegality and illegal use of online content based on six items. Examples of these items include “There are people who do not obey the law, and it would be a loss if I am the only one who follows the law,“ “This society will not change just because one person is more disciplined,” and “My illegal use of online content alone will not cause the copyright holder to suffer a lot.“ Each item was rated on a six-point Likert scale ranging from 1 (*not at all true*) to 6 (*very true*), and Cronbach’s α of these six items was 0.93 in this study.

### Data collection procedures

Prior to data collection, this study was approved by the Institutional Review Board (IRB), and all procedures were performed ethically. Along with written informed consent, they were presented to the respondents online when data were gathered. Participants were informed that they could withdraw at any time while responding to the questionnaire. If they felt discomfort during the survey, they were also informed that debriefing would reduce psychological distress. Furthermore, it was stated that all data obtained anonymously from the survey would be used for research purposes only and kept on an encrypted computer for three years before being discarded.

### Statistical analysis

This study used the IBM SPSS (Statistical Package for the Social Sciences) Statistics for Windows 25.0 and Analysis of Moment Structure (AMOS) 23.0 for statistical analyses. SPSS was used to perform Pearson product-moment correlational analysis and stepwise regression analysis and to calculate skewness and kurtosis of the data to check the condition for parametric statistical analyses.

AMOS was used to perform the path analysis using the maximum likelihood (ML) estimate. The Tucker-Lewis index (TLI), comparative fit index (CFI), absolute goodness-of-fit index (GFI), and root mean square error of approximation (RMSEA) were used to evaluate the goodness-of-fit of the relevant data. An RMSEA of < 0.05 was considered a close fit; < 0.08 was considered to suggest a good model fit, and < 0.10 was considered an acceptable model fit. Moreover, a GFI and CFI larger than 0.90 and a TLI larger than 0.95 indicated a relatively good model fit. A TLI > 0.90 and < 0.95 was considered an acceptable model fit [42].

Bootstrapping was used to verify the causal relationships between variables and analyze the significance of the mediating effect. The moderated TPB model was examined using individual parameters to estimate a single-model effect with two samples. Furthermore, individual parameter differences were examined by estimating the moderating effect of a single model with two different samples. If it was unclear whether there was a moderating effect, the differences between the two models for the two samples were also analyzed. Groups with high and low outcome expectancies and social loafing were divided into medians of 9 and 12, respectively. Therefore, those who scored 10 or more on the items for measuring outcome expectancies were considered to belong to the strong outcome expectancy group, and those who scored 13 or more on the items for measuring social loafing were considered to belong to the strong social loafing group.

## Results

### Relationships between the variables involved in illegal online content use

Table 1 shows the results of the analysis of the relationships between TPB factors, outcome expectancies, social loafing, and illegal online content use among college students. The absolute values of skewness and kurtosis did not exceed 2 for any of the variables, indicating that the variances of all the variables were close to a normal distribution, thus satisfying the condition for conducting parametric statistical analyses [43].

Correlation analysis revealed significant positive relationships between the TPB factors involved in illegal online content use. Attitudes (r = 0.61, *p* < 0.001), subjective norms (r = 0.56, *p*  < 0.001), and perceived behavioral control (r = 0.56, *p*  <  0. 001) were positively correlated with intention to use online content illegally. Attitudes (r = 0.49, *p*  < 0.001), subjective norms (r = 0.50, *p*  <  0.001), and perceived behavioral control (r = 0.56, *p* < 0. 001) were also positively correlated with illegal behavior regarding online content use. Furthermore, intention was closely related to illegal online content use (r = 0.74, *p*  < 0.001), accounting for 54.8% of the variance in illegal online content use.

**Table 1:** The correlational matrix of TPB factors, outcome expectancies, and social loafing for illegal online content use

Variables	1	2	3	4	5	6	7
1. Attitude	1						
2. Subjective norm	0.42***	1					
3. Perceived behavioral control	0.30***	0.50^***^	1				
4. Intention	0.61***	0.56***	0.56***	1			
5. Outcome expectancies	0.48***	0.52***	0.54***	0.69***	1		
6. Social loafing	0.52***	0.42***	0.37***	0.64***	0.63***	1	.
7. Use behavior	0.49***	0.50***	0.56***	0.74***	0.71***	0.58***	1
Mean Standard Deviation	9.26 (3.77)	11.86 (4.21)	10.24 (4.16)	7.21 (3.60)	9.66 (4.32)	13.86 (6.58)	11.77 (4.98)
Skewness	0.05	0.10	0.36	1.10	0.43	0.75	0.66
Kurtosis	-1.29	-0.32	-0.89	0.76	-0.47	0.16	0.35

***p < 0.001

Outcome expectancies were positively correlated with intention (r = 0.69, *p* < 0.001) and behavior (r = 0.71, *p* < 0.001) regarding illegal online content use. This accounted for 50.4% of the variance in illegal behavior regarding online content use. Social loafing was also positively correlated with intention (r = 0.64, *p* < 0.001) and behavior (r = 0.58, *p* < 0.001) regarding illegal online content use.

We conducted a stepwise regression analysis to predict the participants’ illegal use of online content with respect to TPB factors, outcome expectancies, and social loafing (Table 2). Multicollinearity occurs when the tolerance value is lower than 0.1 or 0.2, and variance inflation factor (VIF) values exceed 5 or 10 [44]. Because the tolerance values of predictors in this study approached 0.344–0.608, and the values of VIF approached 1.645–2.906, multicollinearity was not significant. Additionally, the Durbin–Watson statistic in this regression model was 2.054, suggesting that no autocorrelation was detected because it was close to 2.

Stepwise regression analysis revealed that intention (β = 0.74, *p* < 0.001), social loafing (β = 0.37, *p* < 0.001), outcome expectancies (β = 0.14, *p* < 0.001), and perceived behavioral control (r = 0.509, *p* < 0.05) were significant predictors of illegal online content use in this model. These four predictors loaded on illegal online content use (R^2^ = 0.544, *p* < 0.001). Furthermore, intention accounted for the highest amount of variance, followed by social loafing, outcome expectancies, and perceived behavioral control.

**Table 2:** Results of the stepwise regression analysis for TPB factors, outcome expectancies, and social loafing with regard to illegal online content use

Variables	*β*	*t*	*∆R* ^2^	*F*
Intention	0.74	20.91***	0.544	157.02***
Social loafing	0.37	8.33***	0.072	
Outcome expectancies	0.14	3.52***	0.013	
Perceived behavioral control	0.09	2.03*	0.004	

*p < 0.05, ***p < 0.001

### Path analysis of the proposed models

This study presents the proposed models based on the TPB and attempts to determine the optimal model by comparing its goodness-of-fit with that of proposed Model I, which was without the path of perceived behavioral control → use behavior, and of proposed Model II, which added the path of perceived behavioral control → use behavior. The fit index used in this study was (in addition to the commonly used TLI, RMSEA, GFI, and CFI) based on accountability and simplicity.

The χ^2^ value of the proposed Model I was 31.65 (df = 3, p < 0.001), and the GFI was 0.968; the other values were as follows: TLI = 0.964, CFI = 0.880, and RMSEA = 0.161 (Table 3). A significant χ^2^ value indicated that this model could vary depending on sample size. The GFI and TLI indices were found to be greater than 0.90 and fell within the condition of good model fit, but the CFI (below 0.90) and RMSEA (0.10 and above) were outside the required values for acceptable model conditions.

**Table 3:** Comparison of the goodness-of-fit between the proposed models I and II

Model	χ^2^	*df*	GFI	TLI	CFI	RMSEA (90% confidence interval)
Proposed Model I	31.65***	3	0.968	0.964	0.880	0.161 (0.113 ~ 0.214)
Proposed Model II	5.63	2	0.994	0.977	0.995	0.070 (0.000 ~ 0.142)

*** *p* < 0.001

The χ^2^ value of the proposed Model II with the added path of perceived behavioral control → use behavior was 5.63 (df = 2, n.s.), and the goodness-of-fit indices were GFI = 0.994, TLI = 0.977, CFI = 0.995, and RMSEA = 0.070 (Table 3). The χ^2^ value for this model is not statistically significant. Thus, the model with this sample well represented the total population; this means that there is no significant difference between the observed and estimated covariance matrices. The GFI, TLI, and CFI indices were all > 0.95, suggesting an excellent model. Furthermore, the RMSEA was lower than 0.08, indicating a good model. Thus, it was not necessary to compare the model fit with χ^2^ differentiation regarding nested relationships; this indicates that the proposed Model II could be adopted. In short, this study validated the model by adding the perceived behavioral control → use behavior path as a useful TPB to analyze illegal online content use.

**Fig. 2:**
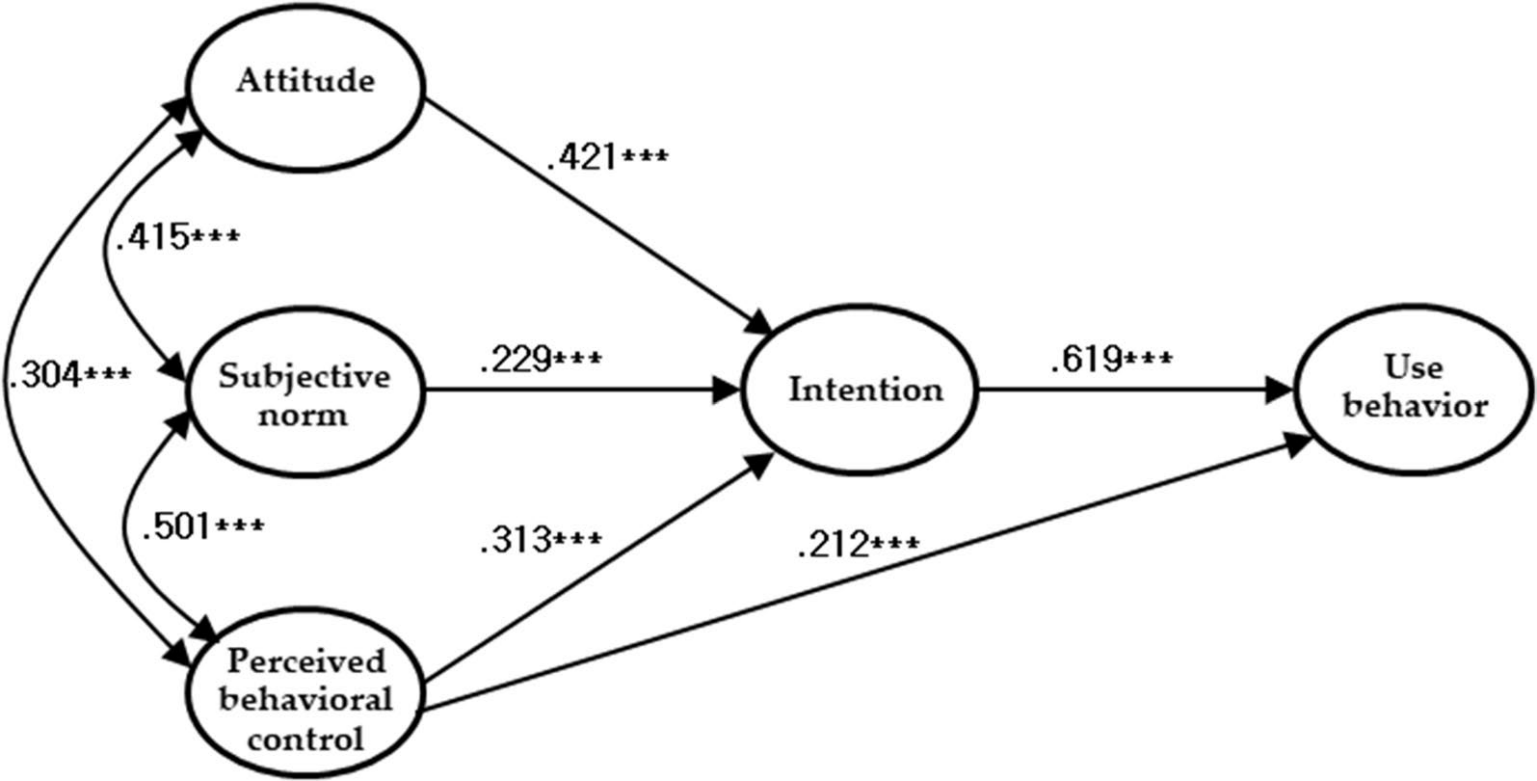
The path map of the proposed Model II of TPB (***p < 0.001)

The path coefficients in the proposed Model II are shown in Fig. 2; Table 4. Regarding each path coefficient in the adopted model that involved illegal use of online content, the results revealed that a more positive attitude predicted a higher likelihood of intention to illegally use online content (β = 0.421, *p* < 0.001), a stronger subjective norm for using illegal online content predicted a greater likelihood of intention to illegally use online content (β = 0.229, *p*  <  0.001), and a higher level of perceived behavioral control predicted a greater likelihood of intention to illegally use online content (β = 0.313, *p* < 0.001). Furthermore, when college students perceived themselves as being able to control their illegal use of online content, they showed a greater tendency to use online content illegally directly (β = 0.212, *p* < 0.001). Of course, a higher likelihood of intention to use online content illegally predicted a greater degree of actual illegal online content use (β = 0.619, *p* < 0.001).

**Table 4:** The estimated parameter values of the adopted model (proposed Model II)

Predictors	Non-standardized weight	Standardized weight	S.E.	C.R.
Attitude → Intention	0.403	0.421	0.037	11.01***
Subjective norm → Intention	0.196	0.229	0.036	5.43***
Perceived behavioral control → Intention	0.223	0.313	0.029	7.78***
Perceived behavioral control → Behavior	0.209	0.212	0.040	5.19***
Intention → Behavior	0.856	0.619	0.057	15.14***

*** *p* < 0.001

Model II was adopted because the direct path of perceived behavioral control to illegal online content use was significant. Furthermore, the analysis of the mediated effects (Table 5) showed that the indirect paths from attitude, subjective norms, and perceived behavioral control through intention to use illegal online content were all significant. Ajzen considered this TPB model with a direct path from perceived behavioral control to behavior (p. 182) [10].

**Table 5:** The mediating effects of the adopted model (proposed Model II)

Predictors	Non-standardized effect	Standardized effect
Attitude → Intention → Behavior	0.345	0.261***
Subjective norm → Intention → Behavior	0.167	0.142***
Perceived behavioral control → Intention → Behavior	0.190	0.194***

***p < 0.001

### The moderating effect of the outcome expectancies on social loafing on the TPB model

This study examined the goodness-of-fit of the TPB model for illegal online content use, with outcome expectancies or social loafing as a moderator.

Table 6 presents the goodness-of-fit comparison results between the adopted TPB model and TPB models with moderating effects. The χ^2^ value of the model where outcome expectancies were included as a moderator was 3.06 (df = 4, n.s.), and the goodness-of-fit indices were GFI = 0.997, TLI = 1.010, CFI = 1.000, and RMSEA = 0.000 (0.000 ~ 0.070). The GFI, TLI, and CFI values were better than those of the original model adopted in this study, and RMSEA was less than 0.05, which was a much better result than that of the original adopted model. However, the χ^2^ value of the model where social loafing was included as a moderator was 22.46 (df = 4, *p* < 0.001), and the GFI for this model approached 0.977; the other values were as follows: TLI = 0.841, CFI = 0.968, and RMSEA = 0.112 (0.070–0.159). Most of these values were worse than those of the original adopted model; in particular, the TLI (below 0.90) and RMSEA (above 0.10) were unacceptable.

**Table 6:** Comparison of the goodness-of-fit with the moderating effects of outcome expectancies and social loafing

Model	χ^2^	*df*	GFI	TLI	CFI	RMSEA (90% confidence interval)
Adopted model	5.63	2	0.994	0.977	0.995	0.070 (0.000 ~ 0.142)
Model with outcome expectancies	3.06	4	0.997	1.010	1.000	0.000 (0.000 ~ 0.070)
Model with social loafing	22.46***	4	0.977	0.841	0.968	0.112 (0.070 ~ 0.159)

***p < 0.001

**Fig. 3:**
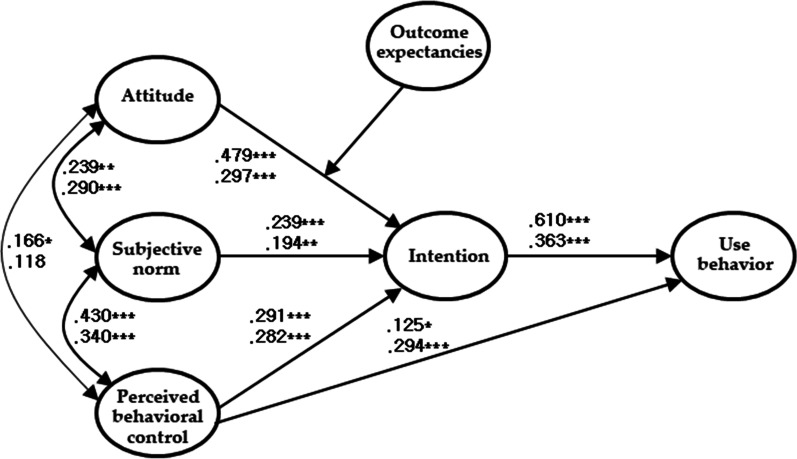
The path map of the model with the moderating effect of outcome expectancies (*p < 0.05, **p < 0.01, ***p < 0.001; the upper part is for those with high outcome expectancies, and the lower part is for those with low outcome expectancies)

To clarify the moderating effect of outcome expectancies on the TPB model, we analyzed the differences between the two models with two samples to elucidate the moderating effects of outcome expectancies on illegal online content use. We found significant differences between the students with high and low outcome expectancies (*p* < 0.001). The normed fit index of the unrestricted and restricted models differed significantly by 0.057, and the critical ratio for the unrestricted model was 5.31, exceeding 1.96, which revealed that the difference between these two groups was also significant at the 0.001 level. The results indicate a difference between the model with high outcome expectancies and that with low outcome expectancies. As shown in Fig. 3, the groups with high outcome expectancies showed a higher coefficient of indirect effect through intention to use behavior, whereas students with low outcome expectancies showed a higher coefficient of the direct path from perceived behavioral control to use behavior.

**Fig. 4:**
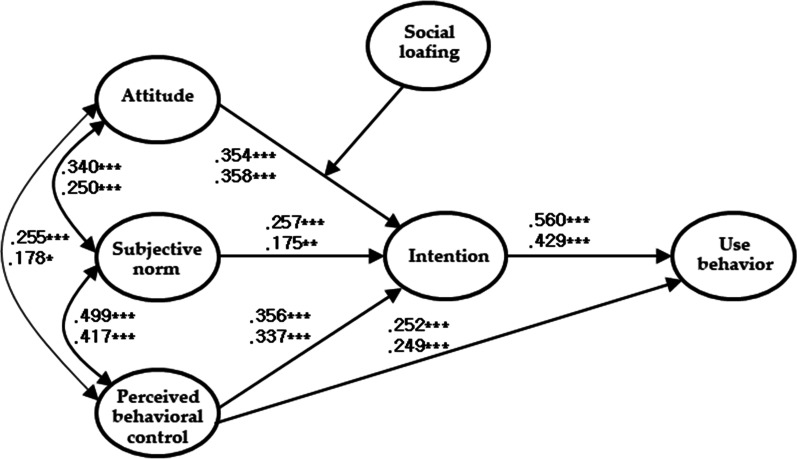
The path map of the model for groups with high and low social loafing (*p < 0.05, **p < 0.01, ***p < 0.001; the upper part is for those with high social loafing, and the lower part is for those with low social loafing)

We can conclude that social loafing has no moderating effect on the TPB model for illegal online content use, as the model fit became unacceptable when social loafing was entered as a moderating variable. Unlike the moderating effect of outcome expectancies, as shown in Fig. 4, there were no significant differences in the coefficients of the direct effect of perceived behavioral control and the indirect effects of attitude, subjective norms, and perceived behavioral control on illegal online content use behavior between the groups with high and low social loafing.

## Discussion

Currently, the necessity of eradicating illegal online content use is emphasized in an era where individuals can produce online content to generate profit, and such content is being consumed through over-the-top services. This study verified models that could predict the illegal use of online content. The applications and implications of this study’s findings are discussed below.

The current study’s correlational analysis found that attitude, subjective norms, perceived behavioral control, and intention regarding illegal online content use, which are TPB variables, were closely related to illegal behavior regarding online content use; thus, the alternative hypothesis H1 was accepted. Many studies [16–18] have shown that perceived behavioral control, among the TPB variables, accounts for the greatest variance in behaviors, even if the accountability of perceived behavioral control for behaviors is greater than that of behavioral intention [14], and that the accountability of perceived behavioral control with regard to illegal behavior regarding online content use was not greater than that of the other TPB variables in this study. Regarding restrained dietary practice, smoking behavior, or smoking cessation [18, 20, 45, 46], perceived behavioral control may be decisive; however, in the case of committed illegal behaviors, the influence of attitude and subjective norms may be as important as that of perceived behavioral control.

In this study, behavioral intention, among the TPB variables, accounted for the greatest variance in illegal online content use. This reiterates Fishbein and Ajzen’s assumption that intention is the strongest predictor of behavior and that intention induces behavior by mediating attitudes and subjective norms [15]. Therefore, in this study, the fit of the TPB model, in which behavioral intention mediates attitudes, subjective norms, and perceived behavioral control with regard to engaging in illegal online content use, included a direct path from perceived behavioral control to illegal use behavior and was found to be satisfactory. However, the fit of the TPB model, which did not include a direct path from perceived behavioral control to illegal use behavior, was not acceptable. In other words, the alternative hypothesis H2a is rejected, and H2b is accepted. This issue was also proposed when Ajzen introduced TPB [10]; thus, perceived behavioral control may have some influence that directly induces behavior, along with the influences that cause behavioral intention.

These results have both policy and educational implications. First, young people should be made aware of the significantly negative social norms regarding illegal online content use by strengthening laws or media campaigns [47]. In addition to the legal punishment for those who illegally use online content, felony punishment for those who provide the devices or networks that supply illegal online content could lower young people’s perceived behavioral control regarding engagement in illegal online content use. Furthermore, it is necessary to provide education in schools, communities, and media so that adolescents can develop negative attitudes toward illegal online content use.

In this study, expectations regarding the outcomes of illegal online content use were closely related to illegal behavior regarding online content use. Outcome expectancies accounted for 50.4% (r = 0.71) of the variance in illegal behavior regarding online content use, far greater than the 31.4% (r = 0.56) accountability of perceived behavioral control regarding illegal use behavior. The concept of outcome expectancies, including considerations of rewards, perceived risks, and perceived sanctions, is a major component of social cognitive theory [28], which Lowry et al. concluded after conducting a meta-analysis, suggesting that it was a determinant for digital piracy [30]. Once again, this study confirmed that outcome expectancies can play an important role in inducing college students to illegally use online content. Thus, this study showed that it is important to strengthen the law through policymaking so that college students can recognize that their legal responsibilities outweigh the financial benefits they can obtain as a means of preventing illegal online content use.

Most importantly, this study found that outcome expectancies could moderate the TPB model for illegal online content use; that is, H3a was accepted. The TPB model for predicting illegal online content use among Korean college students showed significant differences in paths depending on the level of outcome expectancies. Previous studies have found that outcome expectancies moderate the relationships between certain thoughts and behaviors or responses [46, 48]; however, beyond these functions, outcome expectancies can also moderate the model predicting illegal online content use. In the present study, those with strong outcome expectancies showed a higher coefficient of the indirect effect through “intention to illegally use” behavior, whereas those with weak outcome expectancies had a higher coefficient of the direct path from perceived behavioral control to illegal use behavior. This result could be attributed to the fact that outcome expectancies influence behavioral intention to a greater degree than any perceived behavioral control over illegal online content use. This study revealed that outcome expectancies shared 47.6% (r = 0.69) of the variance in behavioral intention, whereas it shared 29.2% (r = 0.54) of the variance in perceived behavioral control for illegal online content use.

However, H3b was not supported. In this study, social loafing did not moderate the TPB model, which predicts illegal online content use. This is inconsistent with the results of a previous study, which found that social responsibility dilution moderates the TPB model for the illegal use of music sources [24]. Although social loafing did not moderate the TPB model, we found that it played an important role in college students’ illegal online content use. This study found that a higher level of social loafing predicted a greater number of college students illegally using online content. Thus, beliefs related to the statements “just one person’s ability to maintain order will not change society” and “just one person’s use of illegal online content will not cause the copyright holders to suffer much damage” could lead to greater illegal use of online content. This result indicates that social responsibility dilution can also be applied to college students’ illegal online content use. In Lim and Suh’s study, the accountability of social responsibility dilution for adolescents’ illegal use of music sources was 12.3% (r = 0.35) [23]. However, the accountability of social loafing with regard to college students’ illegal online content use was 33.6% (r = 0.58) in this study. This suggests that dealing with social loafing—social responsibility dilution—can prevent illegal online content use. In other words, we should first prevent social loafing from permeating society; students should then be educated from primary school to preserve their sense of social responsibility to prevent the illegal use of online content in adolescence and early adulthood.

## Limitations of the study

Despite the implications discussed above, this study had some limitations that should be considered. First, the study sample was not perfectly representative of Korean college students, as they were registered by an online survey research company. However, this study is based on illegal online content use; therefore, it can form an appropriate sample because college students across the country actively use the Internet. Second, although the cause-effect relationship was discussed based on previous studies and logic, causation cannot be definitively concluded based on correlational studies. Finally, because no complete conclusion could be reached based on a single study, the roles of the TPB model and variables from TPB, outcome expectancies, and social loafing in illegal online content use should be explored further to confirm the findings of this study.

## Conclusion

This study validated the usefulness of the TPB model for predicting college students’ illegal online content use and a model including a direct path from perceived behavioral control to illegal use behavior, which was proposed by Ajzen [10]. The TPB model adopted in this study was moderated by outcome expectancy. Furthermore, this study found that outcome expectancies and social loafing could lead college students to illegally use online content. However, the study results should be reaffirmed because this was a single correlational study with participants who did not represent all populations.

## Data Availability

The datasets used and/or analyzed in this study are available from the corresponding author upon reasonable request.

## Abbreviations

TPB: Theory of planned behavior
SNS: Social network services
TRA: Theory of reasoned action
DSPQ: Dilution of Social Responsibility Questionnaire
IRB: Institutional Review Board
SPSS: Statistical Package for Social Sciences
AMOS: Analysis of Moment Structure
ML: Maximum Likelihood
TLI: Tucker–Lewis Index
CFI: Comparative fit index
RMSEA: Root mean square error of approximation
SRMR: Standardized root mean square residual
VIF: Variance inflation factors
NFI: Normed fit index
